# Ex vivo mechanical testing of double strand, braided and knitted polyethylene suture for acute transverse section of the Achilles tendon in a dog model

**DOI:** 10.1186/s13018-025-05947-1

**Published:** 2025-05-30

**Authors:** Manar Y. Abd El-Aziz, Doaa H. Elgohary, Y. A. Abo El Amaim, Elham A. Hassan

**Affiliations:** 1 Clothing and Knitting Industrial Research Department, Textile Research and Technology Institute, National Research Centre, 33 EL Bohouth St. (former EL Tahrir St.)– Dokki, Giza, 12622 Egypt; 2 Spinning and Weaving Engineering Department, Textile Research and Technology Institute, National Research Centre, 33 EL Bohouth St. (former EL Tahrir St.)– Dokki, Giza, 12622 Egypt; 3 Spinning, Weaving and Knitting Department, Faculty of Applied Arts, Beni Suef University, Beni Suef, 62512 Egypt; 4 Department of Surgery, Anesthesiology and Radiology- Faculty of Veterinary Medicine, Cairo University, Giza, 12211 Egypt

**Keywords:** Tendon repair, Tenorrhaphy, Suture, Polyethylene yarn, Animal model

## Abstract

**Background:**

Acute tendon cut represents a great challenge both in human and veterinary medical practice. The current study aimed to compare the ultimate biomechanical properties (tensile strength, elongation, stress load, yield load and break load) of double strand, braided and knitted polyethylene suture in an ex vivo model of acute transverse section of the Achilles tendon in dog model using locking loop suture and three-loop pulley suture.

**Methods:**

A-thirty-six Achilles tendon was transected from 18 dog cadavers. Tendon samples were randomly allocated (6 tendons/group) to be sutured either by double strand, braided and novel knitted formation techniques from polyethylene suture using either three-loop pulley suture or locking loop suture patterns. Biomechanical testing of different yarn for tensile strength, elongation, stress, yield load, break load was performed.

**Results:**

Braided polyethylene sutures demonstrated superior biomechanical properties, showing the highest maximum tension, load, stress, and yield load, while knitted sutures exhibited the greatest strain and elongation due to their looped structure. Despite the knitted yarn’s high elongation, its tensile strength and load-bearing capabilities were significantly lower. Overall, yarn formation had a greater influence on biomechanical performance in association with suturing technique. The three-loop pulley suturing demonstrated significantly improved suturing outcomes.

**Conclusion:**

Both novel knitted, and braided suture structure demonstrated improved biomechanical properties of tendon suturing by increasing the number of strands within the tendon, simplifying the suturing process, reducing the needle passes, and minimizing tendon punctures that may interfere with healing and the overall strength. Suturing technique had a major influence on the biomechanical properties where the three-loop pulley suture demonstrated superior biomechanical properties compared to locking loop suturing.

## Background

Tendon injury represents a great challenge both in human and veterinary medical practice [1–4]. Such injury usually arises following chronic overstretching of the tendon, acute trauma or secondary to laceration or penetrating wound [5]. Tendon injury is usually associated with severe degree of lameness, disability, considerable pain, in addition to the prolonged care and treatment cost [4–6]. The processes of tendon injury, healing, and repair are complex which may span several months for optimum healing and remodeling [4, 7]. Management of acute tendon cuts typically involves primary end-to-end suturing for optimum tendon healing to restore tendon’s function and prevent long-term complications [8–11]. Successful tendon repair necessitates a careful balance between stability and controlled post-operative motion [12–15]. The key factors for optimum stability at the repair site include easy suture placement, minimal bulk, gliding resistance, adequate nourishment, and preservation of the tendon’s blood supply [16].

Despite the ongoing advancement in suture techniques and materials, complications still arise in approximately 15% of cases, with rupture of the tendon at the repair site in almost 5% of sutured tendons [17]. These complications have a significant impact on the quality of life, often necessitating additional surgery and resulting in prolonged or permanent functional deficits. Therefore, continuous research is required to enhance the strength and durability of tendon repairs, aiming to reduce the incidence of repair failures under normal physiological conditions.

Initial fixation strength is a major contributor in successful tendon repair which is mainly influenced by the type of suture material, suturing technique, immobilization and gap formation.

It has been reported that early motion and multistrand core repair are beneficial for the long-term treatment outcome [18–20].

Gap formation has a detrimental effect on healing outcome as it delays healing time, reduces strength at the repair site, increase risk of re-rupture. For successful clinical outcome, tendon suturing should possess sufficient strength to allow for early range of motion, easily to be performed, with minimum damage to tendon vasculature, and should not be cumbersome to ensure seamless tendon gliding. However, as the number of strands crossing the tendon increases, more manipulation is required to insert them into the tendons, necessitating a greater number of knots for tying, ultimately leading to a bulky structure [21].

Failure of sutures to maintain apposition at the cut ends is well characterized using monofilament sutures. Previous studies have indicated the use of mesh suture was designed to decrease suture pull-through, improve tissue hold and facilitate tissue integration into and around the filaments [22].

The use of braided suture materials has gained recent attention to provide a stronger suturing compared to monofilament sutures. The braiding structure of the suture material distributes the tension along the length of the thread, reduce the risk of cutting through delicate tendon tissues during healing process [23]. Knitting of the monofilament as one wale may provide an additional biomechanical support to resist gap formation. The texture of the knitted structure may provide a texture that promote tissue ingrowth to facilitate healing process and reduce the likelihood of gap formation.

Polyethylene is a synthetic non-absorbable suture material produced through various polymerization methods. Polyethylene is commonly used in tendon suturing due to its high tensile strength, minimal tissue reactivity and biocompatibility. It is an ideal option for tendon suturing due to its long-term stability. The smooth surface of polyethylene suture material allows for easy handling [24].

In the current investigation, we developed a novel braided and knitted polyethylene yarn formation to be used in suturing acute Achilles’ tendon of dog cadavers. The study aimed to compare the ultimate biomechanical properties (tensile strength, elongation, stress load, yield load and break load) of double strand, braided and knitted polyethylene suture in an ex vivo model of acute Achilles’ tendon cut in dog model using locking loop suture and three-loop pulley suture. We hypothesized that the braided and knitted structure may have improved biomechanical properties compared to double strand suture.

## Material and method

### Study design

A polyethylene yarn was prepared in a double strand, braided, and knitted suture material. Biomechanical testing of yarn properties was made to evaluate the maximum tension, maximum strain, and maximum load of the three tested polyethylene sutures. An ex vivo model was designed to induce an acute tendon cut within the Achilles tendon in 18 dog cadavers. Tendon cuts were sutured with the three designed suture materials (double strand, braided and knitted polyethylene suture) using two suture patterns (locking loop suture and three loop pulley suture). Biomechanical testing of the sutured tendons was made to evaluate the tensile strength, elongation, stress load, yield load and break load of each group.

### Yarn preparation and characterization

**Fig. 1:**
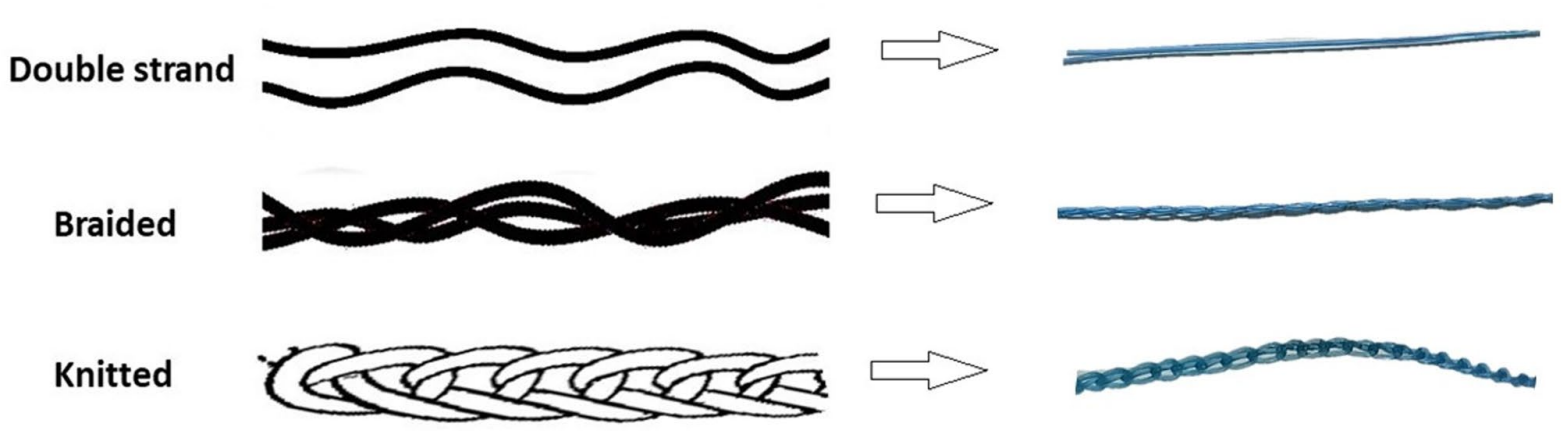
Polyethylene yarn with three different techniques. (**a**) plying by two double strands, (**b**) braiding by three monofilaments, and (**c**) knitting one monofilament as one wale

A monofilament polyethylene yarn manufactured by the United company for plastic industries with a diameter of 250 microns was used. The polyethylene suture was custom-made by three different techniques (plying by two double strands, braiding by three monofilaments, and knitting one monofilament as one wale) to produce three different suture yarns (Fig. 1). This prototype suture was designed to mimic the properties of high-tensile strength clinical sutures while allowing for controlled evaluation of mechanical performance in tendon repair models. Although not commercially available at present, the thread shares structural and compositional characteristics with existing clinical-grade sutures.

Biomechanical characterization of yarn properties was made to determine the maximum tension, maximum strain, and maximum load of the three tested polyethylene sutures.

Biomechanical testing was made at the ambient temperatures (20 °C+/-2) and relative humidity levels (65%+/-2RH) in accordance with LST EN ISO 139 (2006) [25].

A uniaxial testing device (Model 3345, Instron, USA), at room temperature (20 °C+/-2), was used according to the standard methods ASTM D2256 [26] which specifies the method for determining the tensile properties of monofilament, multifilament, and spun yarns.

### Ex vivo tendon suture

An ex vivo model was designed where 36 Achilles tendon samples were transected from 18 dog cadavers that were euthanized for educational purposes at the Department of Surgery, Anesthesiology and Radiology- Faculty of Veterinary Medicine- Cairo University. All study procedures were carried out in accordance with the Guidelines for Animal Care and Use of Faculty of Veterinary Medicine- Cairo University complying with the national and regional guidelines. All procedures were approved by the Institutional Animal Care and Use Ethical Committee (IACUC) of Faculty of Veterinary Medicine- Cairo University. All experimental procedures were done in accordance with ARRIVE guidelines.

**Fig. 2:**
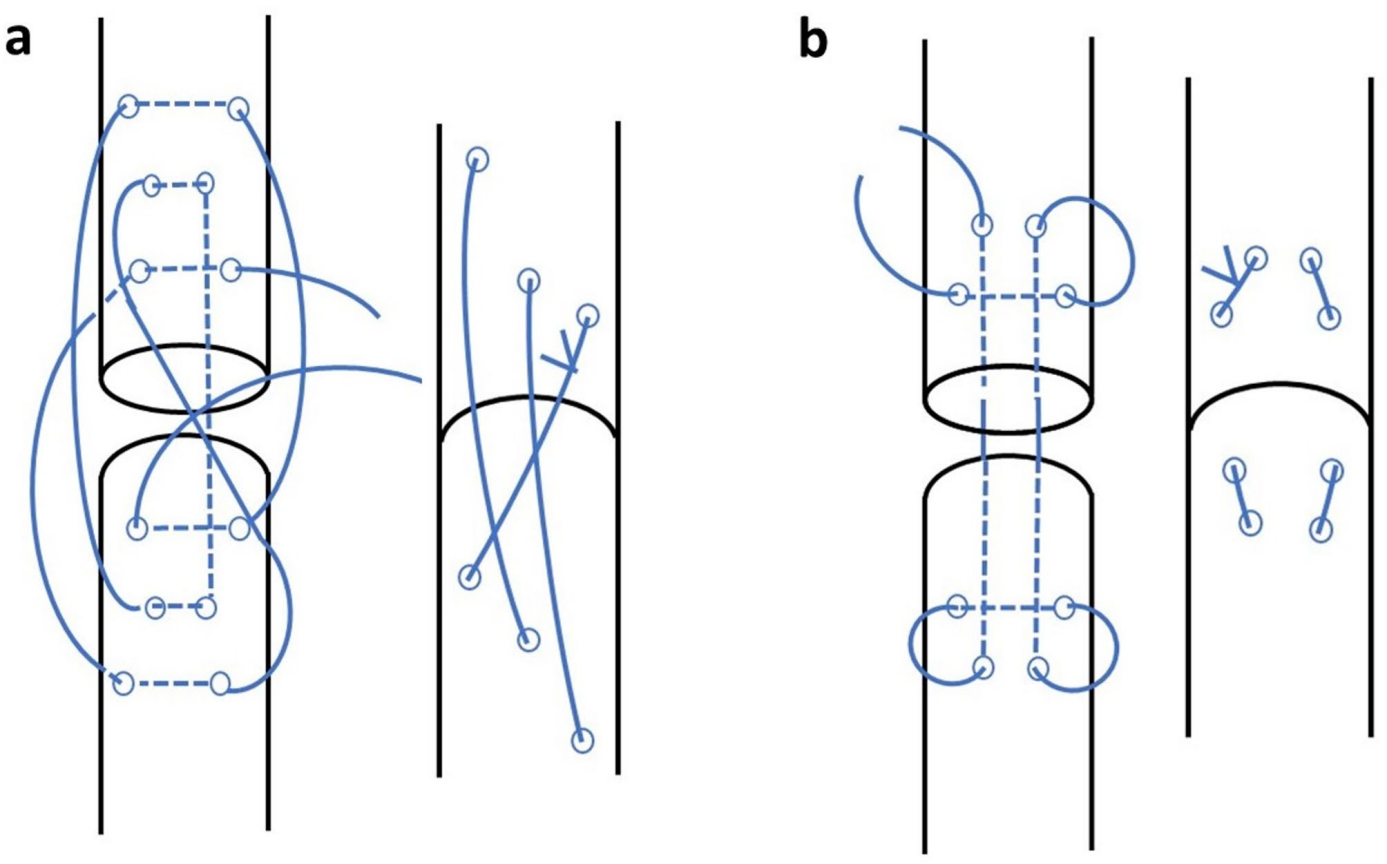
Schematic illustration of tendon suturing technique using three loop pully suture (**a**) or locking loop tendon suture (**b**)

Just following humane euthanasia, the Achilles tendon was exposed, tenotomy was made at middle of the Achilles tendon using a sharp scalpel blade. Tendons were randomly allocated (6 tendons/group) (Table 1) to be sutured using either double strand, braided, knitted polyethylene suture using either locking loop suture or three loop pully suture pattern (Fig. 2).

**Table 1:** Samples specification

Yarn material	Yarn count	Yarn Suture formation	Suture pattern
**Polyethylene (PE)**	250 microns	Double strand	Locking loop suture
			Three-loop pully suture
		Braided strand	Locking loop suture
			Three-loop pully suture
		Knitted strand	Locking loop suture
			Three-loop pully suture

Just following tendon suturing, tendons were transected from its origin and insertion, labelled, and sent for blind biomechanical testing.

### Biomechanical testing of sutured tendons

**Fig. 3:**
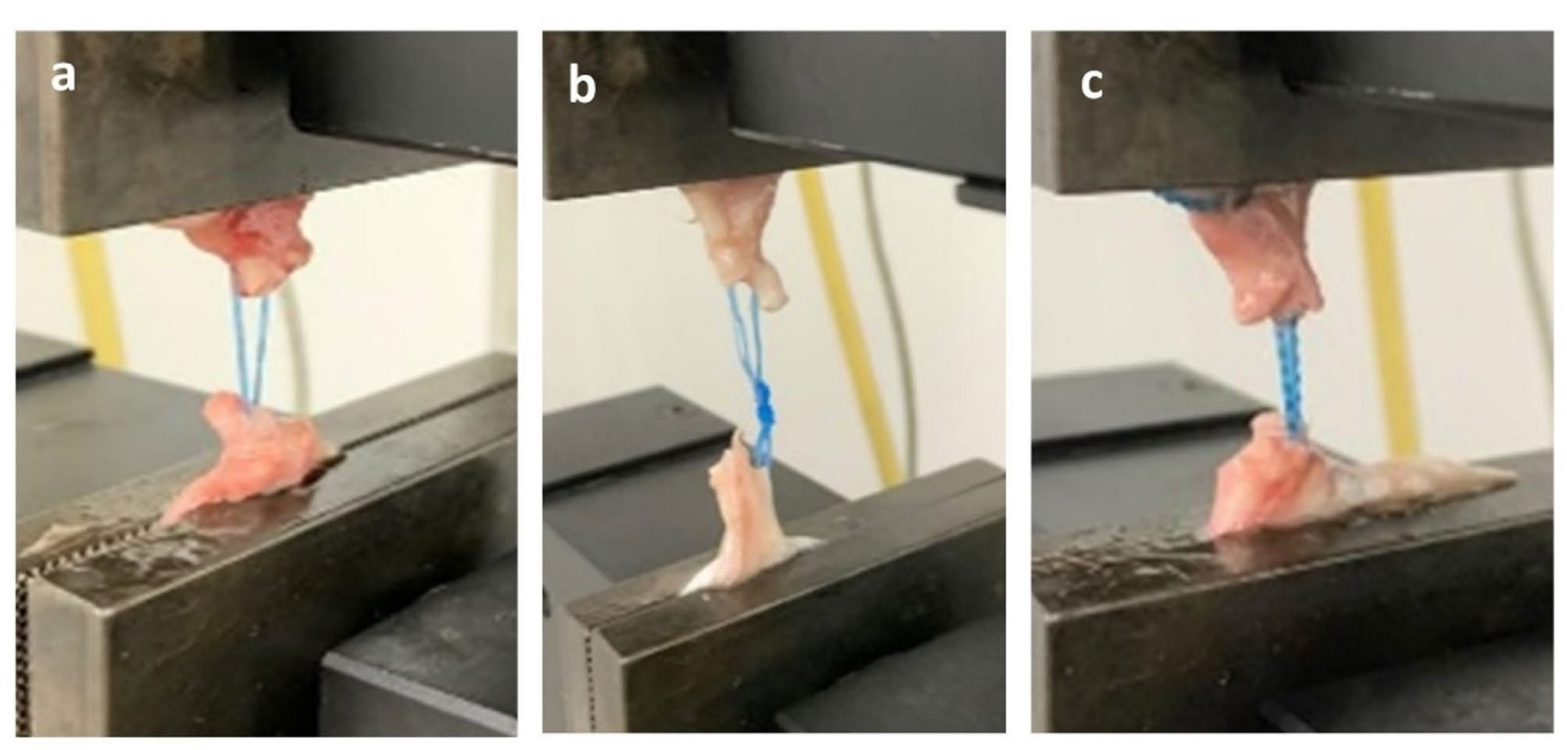
Representative failure modes observed during tensile testing. (**a**) Locking loop suture using double strand polyethylene: failure occurred primarily due to thread breakage at the knot site. (**b**) Three-loop pulley suture using braided polyethylene: failure was caused by pull-out of the thread from the tendon. (**c**) Knitted thread suture: failure occurred at varied locations along the thread, including sites away from the knot, suggesting that internal stress concentration at contact points within the knitted structure contributed to breakage

All tendon samples were preconditioned at the required ambient temperatures (20 °C+/-2) and relative humidity levels (65%+/-2RH) in accordance with LST EN ISO (LST EN ISO, 2006) [25]. A uniaxial testing device (Model 3345, Instron, USA), at room temperature (20 °C+/-2), was used according to the standard methods ASTM D2256 [26]. The sewn tendons were positioned in a vertical position (Fig. 3) to ensure that samples had the same resting length, a preload of 2 N was used. The system was calibrated to enable a constant starting point among independent measures once this preload was obtained. The synchronization of the load was made possible via an automated trigger mechanism. Once the force applied reached 800 N or the point of failure, the sewn tendon samples were distracted at a rate of 20 mm/min, this point was chosen because it represented a two-fold increase in the theoretical force of 400 N [27]. The load (measured in Newtons) and displacement (measured in millimeters) variables of interest were both examined. As yield load was defined as the point at which the build underwent nonlinear deformation, yield, peak, and failure loads were also noted. The maximum force recorded during each test, known as the peak load. The load at which the construct failed or the load at which the load-displacement curve dropped by more than 50% was referred to as the failure load. Suture rupture, suture pull-through, or distant tissue failure were all considered causes of construct failure.

### Statistical analysis

Data were tabulated and expressed as mean ± SD. Normality of distribution was tested using Kolmogorov-Smirnov test. A one-way analysis of variance (ANOVA) was run to determine significant difference among the three tested yarn samples. A Two-way ANOVA was used to statistically analysis the biomechanical impact of three yarn samples by the two sutures techniques on tendon samples. When statistically significant differences were detected, a Tukey Honest Significant Difference (Tukey HSD) test was used for pairwise comparison among different groups. Data were considered statistically significant when the *P* value < 0.05. Data were analyzed using Statistical Package for Social Sciences software (SPSS^®^ Inc. Version 22 for Windows, IBM Corporation, NY, USA).

## Results

### Ex vivo tendon suturing

Tendon suturing was feasible using all polyethylene yarn formation techniques (double strand, braided and knitted polyethylene), using both locking loop and three-loop pulley suturing. Although double strand and braided polyethylene yarns demonstrated easier handling during suturing, the knitted polyethylene yarn demonstrated more secure knot tying during suturing. Subjectively, the three-loop pulley suture was perceived to offer better alignment of the severed tendon ends with minimal distortion of the tendon structure.

### Biomechanical testing of yarn properties

#### Maximum tension property

The mean maximum tension of yarn properties was significantly higher in double strand and braided polyethylene suture compared to the knitted polyethylene.

The braiding structure seems to strengthen the yarn tension despite the knitting structure, the yarn may subject to yarn deformation which may weaken yearn strength as indicated by the decreased maximum tension properties of the knitted yarn.

#### Maximum strain property

The mean values of maximum strain demonstrated that knitted yarn achieved the highest value compared to double strand and braided yarns. No significant difference was recorded in the maximum strain of the double strand and braided yarns. Disparities between statistical significance of tension and strain properties of different yarn are predictable as the strain property is inversely proportional to the tension property.

#### Maximum load property

The braided yarn had the highest mean maximum load properties compared to double strand and knitted yarns. It is obvious that the braiding technique increases the yarn load. Biomechanical testing of different yarn materials is demonstrated in Table 2.

**Table 2:** Biomechanical testing of double strand, braided, and knitted polyethylene suture

Yarn Properties	Yarn Formation Techniques			*P*-value
	Double Strand	Braided	Knitted	
**Max. Tension**	3.46 ^b^ ± 0.26	3.46 ^b^ ± 0.21	1.18 ^a^ ± 0.11	0.000
**Max. Strain**	29.30 ^a^ ± 5.20	35.80 ^a^ ± 10.55	84.05 ^b^ ± 3.53	0.000
**Max. Load**	68.11 ^a^ ± 5.15	137.79 ^b^ ± 50.46	56.68 ^a^ ± 5.08	0.001

Identical superscript letters indicate no statistical significance (*P* > 0.05). Different superscript letters indicate statistical significance (*P* < 0.05)

### Biomechanical testing of sutured tendons

#### Failure modes

Across both suturing techniques, the three primary modes of failure observed were thread breakage, thread pull-out from the tendon, and tendon rupture. In the locking loop suture group, failure occurred most frequently due to thread breakage, typically near the knot. This suggests that the locking mechanism functioned effectively, transferring load directly to the suture material. In contrast, the most common mode of failure in the three-loop pulley suture was pull-out of the suture from the tendon, indicating that the grasping (non-locking) nature was less effective in securing the thread within the tendon. Tendon rupture occurred rarely and did not represent a predominant failure mode in either group.

In the group using knitted threads, thread breakage occurred at various points along the thread, not consistently at the knots. This differs from the failure pattern observed with braided sutures, where failure most often occurred at or near the knot. The variable breakage sites in the knitted group suggest that the increased number of inter-fiber contact points within the knitted architecture may serve as local stress concentrators, leading to distributed failure locations.

#### Tensile strength property

Biomechanical testing of Achilles tendon sutured by braided and double strand polyethylene suturing demonstrated the highest tensile strength compared to knitted yarn. As the braiding structure for yarn supported the strength of yarn, while knitted structure losing its strength during the formation of loop.

Yarn formation had a significant effect of the tensile strength of the sutured tendon (*P* = 0.014). while tendon suturing technique did not have a significant effect on the tendon’s tensile strength (*P* = 0.201).

Tukey HSD test demonstrated no significant differences in the tensile strength between double strand and knitted, double strand and braided polyethylene suture (*P* = 0.098 and 0.484 respectively). While there was a significant difference between knitted and braided yarn (*P* = 0.012).

#### Elongation property

Tendon samples sutured by knitted and braided polyethylene suture had the highest elongation (184.25 and 180.6%) properties compared to double strand yarn (88.72%).

This property can be explained as the elongation is inversely proportion with tensile strength. As the tensile strength increased, the elongation decreased.

Two-way ANOVA analysis demonstrated statistically significant differences in elongation properties among tendons sutured with different yarns (*P* = 0.011). Suture pattern had a statistically significant effect in elongation (*P* = 0.006). Tukey HSD demonstrated differences in elongation among double strand, braided (*P* = 0.023), and double strand, knitted (*P* = 0.018) yarns. While no significant differences were recorded in elongation properties of tendons sutured by braided and knitted yarn (*P* = 0.992).

Comparing the two suturing techniques, the one-way ANOVA analysis demonstrated that locking loop suturing for locking loops had a significant difference in elongation between double strand and knitted- braided- double strand polyethylene yarns (*P* = 0.000 and *p* = 0.009 respectively). While no significant differences were recorded between braided and knitted yarns (*P* = 0.096). Additionally, no significant differences were recorded among tendons sutured by the three-loop pulley suture using different yarns.

#### Stress property

Tendons sutured by braided yarns had the highest stress properties (0.90 Kgf/mm^2^) followed by knitted (0.57 Kgf/mm2) and double strand yarn (0.48 Kgf/mm^2^). As the braiding structure increases the stress of the sutures. The compact structure of braiding structure on the other the knitting structure loses its strength due to the formation of loop which decrease its stress, while double strand recorded the lowest stress according to its loosely structure.

Tendon suturing technique did not have a significant effect on stress properties (*P* = 0.408) while yarn formation had a significant effect (*P* = 0.046). Tukey HSD analysis demonstrated significant differences in stress properties in tendons sutured by between double strand and braided yarns (*P* = 0.048) with no significant differences among tendons sutured by double strand and knitted (*P* = 0.849) and braided and knitted yarns (*P* = 0.124).

No significant differences were recorded in stress properties of tendons sutured locking loop and three loop pulley suture patterns.

#### Yield load property

The yield load properties demonstrated that tendons sutured with braided polyethylene yarn had the highest yield load property (13.07 Kgf) followed by double strand (8.58 Kgf) and knitted yarns (6.77 Kgf). As the braiding structure keeps its strength as a result of its compact structure, while the double strand and knitted structure lose their strength, the double strand as result of it lose structure on the other hand the knitted structure lose its tenacity due to the formation of loop.

Tendon suturing technique did not have a significant effect on yield load properties (*P* = 0.108), while yarn formation had a significant effect (*P* = 0.009).

Tukey HSD testing demonstrated no significant differences in yield load properties among double strand-braided (*P* = 0.056) and double strand-knitted (*P* = 0.564) yarns. While a statistically significant difference was recorded between tendons sutured by braided and knitted yarns (*P* = 0.009).

No significant differences were recorded in yield properties in tendons sutured by double strand, braided and knitted yarns using the locking loop tendon suturing. While using the three-loop pulley suturing, significant differences were recorded in yield load properties between double stranded - braided (*p* = 0.010) and braided - knitted (*P* = 0.003), but not between double strand - knitted yarn (*P* = 0.510).

#### Break load property

The mean value of breaking load revealed no significant differences among the three yarn formation techniques (*P* = 0.600). No significant difference in between the samples (double strand - braided), (braided - knitted) and (double strand - knitted) at *P*-value (0.876, 0.854, 0.571 respectively).

On the other hand, suturing technique had a significant effect on break load (*P* = 0.011). Tendons sutured with three-loop pulley suture using the knitted polyethylene yarn had the highest break load (6.23 Kgf) compared to the locking loop suturing technique (2,92 Kgf).

**Fig. 4:**
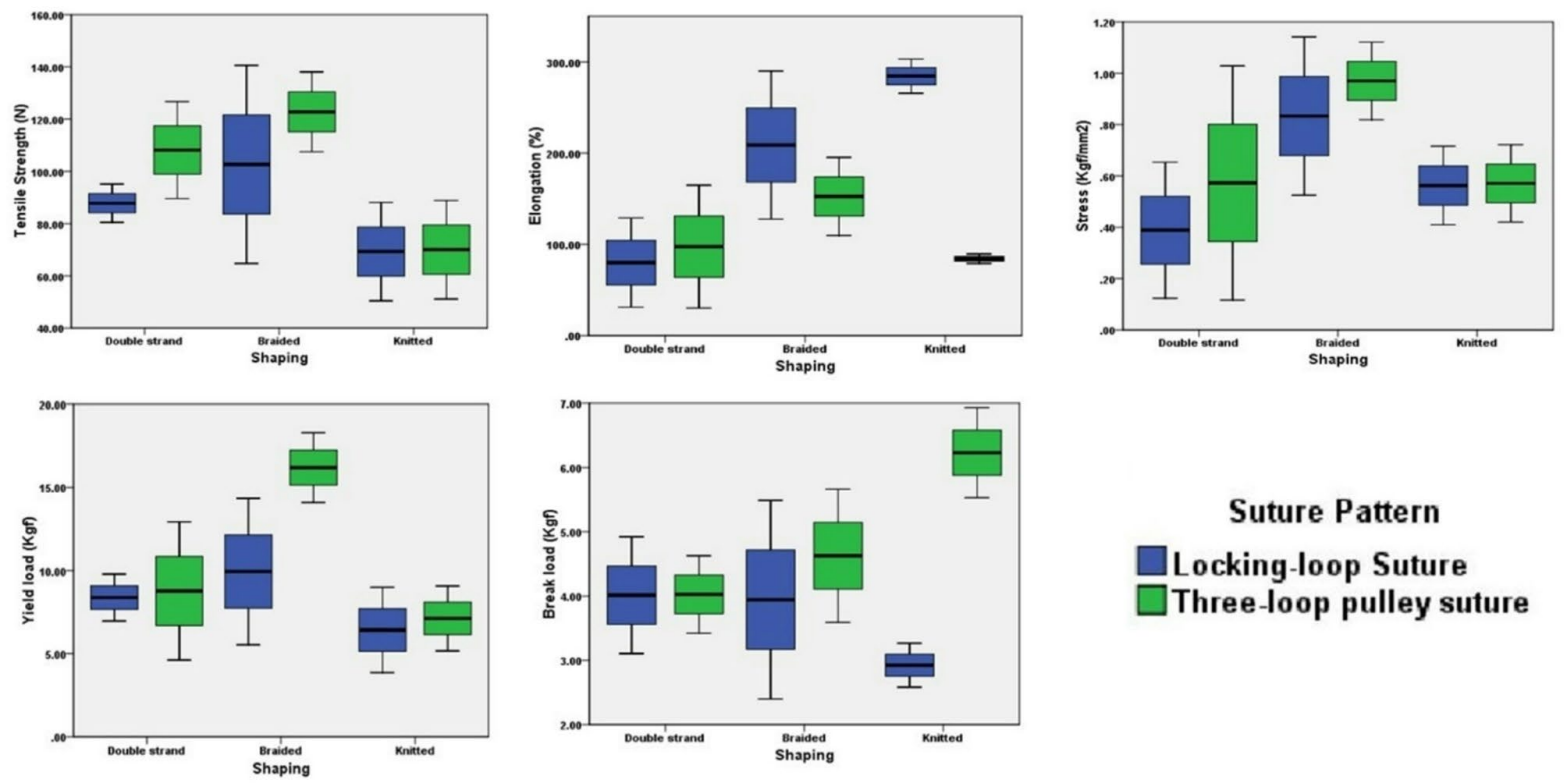
Biomechanical testing including the tensile strength (N), elongation (%), stress (Kgf/mm^2^), yield (Kgf), and break properties (Kgf) of tendon samples sutured by double strand, braided, and knitted polyethylene sutures using locking loop and three-loop pulley suture

Biomechanical testing of the different yarn formation, and suturing techniques is demonstrated in Table 3; Fig. 4.

**Table 3:** Biomechanical testing of sutured tendon properties

Sutured Tendon Properties	Suture pattern	Yarn Formation Techniques			Mean	F-value	*P*-value
		Double Strand	Braided	Knitted			
		Mean ± S.D.					
**Tensile Strength (N)**	**locking loop suture**	87.82^a^ ± 7.32	102.66 ^a^ ± 37.98	69.27 ^a^ ± 18.86	86.58	1.828	0.201
	**Three Loop pully suture**	108.16 ^a^ ± 18.55	122.79 ^a^ ± 15.29	69.99 ^a^ ± 18.89	100.32		
	**Mean**	97.99^ab^	112.72^b^	69.63 ^a^			
	**F-value**	6.198					
	**P-value**	0.014					
**Elongation (%)**	**locking loop suture**	79.94 ^a^ ± 48.87	208.85 ^ab^ ± 81.15	284.35 ^b^ ± 18.65	191.05	10.908	0.006
	**Three Loop pully suture**	97.50 ^a^ ± 67.3	152.50 ^a^ ± 42.80	84.14 ^a^ ± 5.20	111.38		
	**Mean**	88.72^a^	180.68^b^	184.25^b^			
	**F-value**	6.720					
	**P-value**	0.011					
**Stress (Kgf/mm** ^**2**^ **)**	**locking loop suture**	0.39 ^a^ ± 0.27	0.83 ^a^ ± 0.31	0.56 ^a^ ± 0.15	0.60	0.735	0.408
	**Three Loop pully suture**	0.57 ^a^ ± 0.46	0.97 ^a^ ± 0.15	0.57 ^a^ ± 0.15	0.70		
	**Mean**	0.48^a^	0.90^b^	0.57^ab^			
	**F-value**	4.029					
	**P-value**	0.046					
**Yield Load (Kgf)**	**locking loop suture**	8.38 ^a^ ± 1.41	9.94 ^ab^ ± 4.41	6.43 ^a^ ± 2.57	8.25	3.022	0.108
	**Three Loop pully suture**	8.77 ^a^ ± 4.16	16.19 ^b^ ± 2.1	7.12 ^a^ ± 1.95	10.70		
	**Mean**	8.58 ^ab^	13.07^b^	6.77^a^			
	**F-value**	7.063					
	**P-value**	0.009					
**Break Load (Kgf)**	**locking loop suture**	4.01 ^ab^ ± 0.91	3.94 ^ab^ ± 1.55	2.92 ^a^ ± 0.34	3.63	9.147	0.011
	**Three Loop pully suture**	4.02 ^ab^ ± 0.60	4.63 ^ab^ ± 9.81	6.23 ^b^ ± 0.70	4.96		
	**Mean**	4.02^a^	4.29^a^	4.58^a^			
	**F-value**	0.533					
	**P-value**	0.600					

Identical superscript letters indicate no statistical significance (*P* > 0.05). Different superscript letters indicate statistical significance (*P* < 0.05)

## Discussion

The present study demonstrated that the use of braided and knitted polyethylene suture resulted in significantly improved biomechanical properties of tendon suturing by increasing the number of strands within the tendon, simplifying the suturing process, reducing the needle passes, and minimizing tendon punctures that may interfere with healing and the overall strength. Suturing technique had a major influence on the biomechanical properties where the three-loop pulley suture demonstrated superior biomechanical properties compared to locking loop suturing.

Tendons are the functional link between the dynamic musculature and the static bones, transferring the force of muscle contractions to the skeletal system, ultimately enabling movement [6]. The Achilles tendon, is the largest and strongest tendon in the body, is involved in approximately 50% of all sports-related injuries [28]. Biomechanical testing of Achilles tendon rupture in dogs is a well-established model to evaluate suture material and suturing technique [29, 30].

The current study demonstrated that doubling and braiding polyethylene suture was associated with increased tensile strength, decreased elongation and (strain) property with increased maximum load. It has been reported that an ideal suture material should have a high tensile strength to withstand the forces exerted on the tendon during movement and to prevent rupture of the sutures, flexible enough to allow for natural movement without stress concentration [31]. In addition, suture material should not elicit inflammatory response or cause tissue damage which helps to promote healing and reduce the risk of complications. All these factors have been reported in polyethylene suture suggesting its suitability to be an ideal suture for tendon repair [32, 33].

Factors that may influence the failure mode of ex vivo biomechanical testing may include test condition, suture placement, differences in substance resistance to pull-out, or suture itself. All these variables were controlled in the present study where the yarn suture was the main determinant factor to record the findings. The differences in failure modes between the two suture techniques reflect their underlying biomechanical designs. The locking loop suture incorporates a true locking configuration, engaging the tendon with greater security. When this locking structure is effective—consistent with recommendations by Hatanaka et al. (1999) [34] that approximately 30% of the tendon’s cross-sectional area should be involved—the applied force is borne primarily by the suture thread. Consequently, failure tends to occur through thread breakage, usually at or near the knot, and reflects the material strength of the suture rather than slippage or displacement. On the other hand, the three-loop pulley suture operates through a grasping mechanism, with less consistent tendon engagement. In this group, failure was predominantly due to suture pull-out, indicating reduced resistance to longitudinal motion under load. These observations are in line with previous findings (Yotsumoto et al., 2005) [35] regarding the importance of locking structures and knot positioning for optimal load distribution and repair strength.

In addition, the current study did not include polyethylene yarn produced by different manufacturers. However, the study included the same polyethylene monofilament that was either doubled in strand, braided, or knitted where the yarn formation technique was the main determinant factor for biomechanical testing. The use of this custom-made polyethylene suture allowed for preliminary testing of a prototype material designed for potential surgical application. While this custom-made polyethylene suture is not yet commercially available, its composition and performance are representative of polyethylene sutures currently used in clinical practice. Future studies will involve direct comparisons with commercially available suture products to further validate these findings and support clinical translation.

As the strength of tendon repairs is influenced by the size of the core suture [36] and the number of suture strands at the repair site [37], advanced tenorrhaphy techniques have led to increased core suture strands for optimum results [38]. The current investigation aimed to double the strand, braid, and knit the suture material to achieve the maximum strength of the sutured tendon by increasing the number of the strand within the tendon core with minimum tissue damage without any bulky suture. Doubling, braiding, and knitting polyethylene suture effectively increased the number of strands within the tendon, simplified the suturing process, reduced the needle passes, and minimized tendon punctures that may interfere with healing and the overall strength. Our study was designed to increase the number of core strands without increasing the points of entry at the small tendinous tissue. It has been reported that distributing the overall load of the suture-tissue interface among multiple core strands increasing surface area which reduces the force applied at each point, resulting in decreasing the suture pull-through [39].

Knitting the polyethylene yarn into a one wale with continuous square configurations was designed to provide a large surface area with a microporous structure which thought to enhance fibrovascular repair and encapsulation of individual filaments [40]. It was clearly obvious during suturing and biomechanical testing that the knitting structure had resist suture pull-through, which is of the utmost importance to resist gap formation with the dynamic tensile force which is the main cause of repair failure with the standard monofilament suturing. The evenly distributed squares on the knitted suture may provide a unidirectional anchoring across the suture trajectory, thus it did not require complex knotting for tissue grasping and knot securing. The knitting structure provided a homogenous and steady friction against slippage throughout suture trajectory in the opposite direction of the suture introduction.

Biomechanical testing revealed that the knitting structure was found to endure higher loads before failure, especially with using three-loop pulley suturing. Similar results were previously reported using suturable mesh in a cadaveric model of flexor tendon cut [41]. Such criteria along with the increased elongation property, recommend knitted suturing with three three-loop pulley suturing to be an ideal suturing resisting gap formation and withstand early mobilization for early rehabilitation protocols to before significant peri-tendinous adhesion can form and avoid joint stiffness. Additionally, the knitting structure increased the surface area and the size of the suture-tissue interface, lowering the acute force at the interface, reducing the possibility of tearing for a single core strand.

In terms of tensile strength, braided polyethylene sutures achieved a mean value of 112.72 N (± 15.29), significantly higher than double strand (97.99 *N* ± 7.32) and knitted sutures (69.63 *N* ± 18.86), regardless of the suture pattern. These tensile strengths were closely comparable with the findings of Dogar et al., 2024 [24] using the commercially available polypropylene, polyester, and ultra-high molecular weight polyethylene sutures (98.5 ± 5.7, 112.3 ± 6.2, and 134.6 ± 11.2 N respectively) in similar ex vivo ovine tendon suturing models. The knitted polyethylene sutures, however, exhibited a lower tensile strength (69.63 N), reflecting their higher elongation values, which were 284.35% (± 18.65) for the locking loop suture pattern. The current study revealed that braided polyethylene sutures demonstrated the highest yield load (16.19 Kgf ± 2.10) in the three-loop pulley suturing technique, which suggests enhanced resistance to early deformation under loading conditions. In contrast, knitted sutures had a lower yield load (7.12 Kgf ± 1.95) despite higher elongation. Double strand polyethylene sutures performed similarly to knitted sutures (8.77 Kgf ± 4.16). These results are in agreement with Radovanović et al., 2022 and Walton et al., 2024 [42, 43] who reported that higher yield loads are crucial for better tendon repair integrity, reducing the risk of suture failure in high-load conditions.

The elongation data further confirmed the distinct mechanical properties of the polyethylene suture variants. Braided and knitted sutures (180.68% ± 42.80 and 184.25% ± 18.65 respectively) exhibited significantly increased elongation properties compared to double strand sutures (88.72% ± 48.87) suggesting that braided and knitted polyethylene sutures offer better control over gap formation and their suitability to be used in areas of dynamic movement to provide a potentially more stable repair under cyclic loading which is crucial for long-term tendon function [44]. While knitted sutures showed an improved break load (6.23 Kgf ± 0.70) in the three-loop pulley suture pattern compared to other configurations, this performance was accompanied by increased elongation, which may compromise the long-term mechanical integrity of the tendon repair [45]. Braided polyethylene sutures exhibited optimal properties, with a mean break load of 4.29 Kgf ± 0.70, suggesting that a balance between high tensile strength and controlled elongation is key for successful tendon repair.

The use of the three-loop pulley suture and locking loop suture has been advocated to repair transected tendons in dogs [29, 46]. The three-loop pulley suture demonstrated superior biomechanical properties compared to the locking-loop suture. Similarly, the present study demonstrated a higher tensile strength of tendons sutured by three-loop pulley suture compared to locking loop suturing could be explained as with using the 3-loop pulley suture, there are a 6 strands of suture material crossing the anastomosis site, while the locking-loop suture only 2 strands are crossing the site. Increasing the number of suture strands across the anastomosis site has been linked to higher tensile strength in tendon repairs [47]. Doubling the strand, braiding, and knitting the yarn additionally doubled the number of yarns in each suture strand for stronger suture and increased resistance to gap formation.

The improved alignment and minimum distortion of the tendon ends sutured by three-loop pulley suture are consistent with previous findings comparing the three-loop pulley suture with locking loop suture in the equine tendon [48]. The three-loop pulley suture had an increased resistance to gap formation during tensile loading. It was clearly obvious during suturing that the three-loop pulley suture had a better apposition of the cut ends compared to locking loop suturing. Similar findings were previously reported in equine tendon suturing [48]. Three-loop pulley suture necessitated a greater force to generate gap prior to failure compared to locking loop suturing.

On the other hand, the locking-loop patterns had resulted in increased gap formation upon failure in comparison to the 3-loop pulley pattern as demonstrated in significant increase in elongation properties of the braided and knitted polyethylene suture.

In conclusion, improved biomechanical properties of tendon suturing could be achieved by increasing the number of strands within the tendon through braiding and knitted suturing. Braided polyethylene sutures, particularly when used with the three-loop pulley suturing technique, provide superior mechanical performance in terms of tensile strength and yield load, offering a promising alternative for tendon repair, particularly in high-load-bearing tendons. Suturing technique had a major influence on the biomechanical properties where the three-loop pulley suture demonstrated superior biomechanical properties compared to locking loop suturing.

Limitations of the current investigation may include the absence of in vivo study to document tendon healing following suturing. Future studies should be directed towards application of different yarn formation techniques on animal model to document efficacy of healing and long-term efficacy.

## Data Availability

No datasets were generated or analysed during the current study.
